# Effect of Chromium Adhesion Layer Thickness on Contact Resistance and Schottky Barrier Characteristics in WSe_2_ Field-Effect Transistor

**DOI:** 10.3390/nano15181413

**Published:** 2025-09-13

**Authors:** Sung-Ha Kim, Seong-Yeon Lee, Tae-Jeong Kim, Kwangseuk Kyhm, Kenji Watanabe, Takashi Taniguchi, Ki-Ju Yee

**Affiliations:** 1 Department of Physics, Chungnam National University, Daejeon 34134, Republic of Korea; ksh533123@gmail.com (S.-H.K.); bampy99@naver.com (T.-J.K.); 2 Institute of Quantum Systems, Chungnam National University, Daejeon 34134, Republic of Korea; 3 Institut Néel, University Grenoble Alpes, 38000 Grenoble, France; seong-yeon.lee@neel.cnrs.fr; 4 Department of Opto & Cogno Mechatronics Engineering, RCDAMP, Pusan National University, Busan 46241, Republic of Korea; kskyhm@pusan.ac.kr; 5 Research Center for Electronic and Optical Materials, National Institute for Materials Science, 1–1 Namiki, Tsukuba 305–0044, Japan; watanabe.kenji.aml@nims.go.jp; 6 Research Center for Materials Nanoarchitectonics, National Institute for Materials Science, 1–1 Namiki, Tsukuba 305–0044, Japan; taniguchi.takashi@nims.go.jp

**Keywords:** contact resistance, WSe_2_ field–effect transistors, transfer length method, Schottky barrier height, image force lowering

## Abstract

While metal adhesion layers are commonly used in the fabrication of field–effect transistors (FETs) based on two-dimensional (2D) materials, the impact of adhesion layer thickness on device performance remains insufficiently explored. In this study, we systematically investigate how the thickness of a Cr adhesion layer influences the contact resistance and Schottky barrier characteristics of multilayer WSe_2_ FETs. Contact resistance results, extracted via the transfer length method for Cr thicknesses of 1 nm, 4 nm, and 7 nm, reveal that thicker Cr layers (4 nm and 7 nm) result in significantly lower resistance (<200 kΩ·μm) compared to the much higher resistance (6.6 MΩ·μm) observed with 1 nm Cr thickness. Temperature–dependent transport measurements and Arrhenius analysis further indicate a reduction in Schottky barrier height with increasing Cr thickness, implying improved carrier injection. These results specifically demonstrate how the commonly used Cr adhesion layer thicknesses of at least 4 nm increase the electrical performance of WSe_2_–based devices.

## 1. Introduction

Over the past several decades, two–dimensional (2D) materials such as tungsten diselenide (WSe_2_) and molybdenum disulfide (MoS_2_) have garnered significant attention as promising candidates for next–generation electronic and optoelectronic devices due to their unique electrical and optical properties. Applications of 2D materials include various optoelectronic devices such as 2D material–based photodetectors, memory devices, gas sensors, and field–effect transistors (FETs). The fundamental architecture of these devices typically features metallic source and drain electrodes in contact with a 2D semiconductor channel that allows to evaluate electrical performance. Utilizing this 2D electronic platform, extensive research has been conducted to explore the interesting electrical properties of 2D materials, including high carrier mobility, tunable bandgaps, high on/off current ratio, and low power consumption [1,2,3,4,5,6,7]. Nevertheless, realizing higher performance 2D electronic devices remains a technical challenge. Particularly, one of the primary limiting factors playing a critical role in device performance is the contact resistance at the interface between the metal electrodes and the 2D semiconductor channel [8,9].

Contact resistance at the metal–semiconductor interfaces is influenced by several factors, including the choice of contact metal, resist residues left from fabrication processes, adsorption of ambient molecules such as water and oxygen, and trapped charges at the interface. Among these, selection of the electrode metal has a significant effect on charge injection through Schottky barrier formation, metal–induced gap states, and interfacial contamination [10,11,12,13,14,15]. Specifically, Au, a commonly used electrode metal, exhibits relatively weak van der Waals bonding with both SiO_2_ and WSe_2_ surfaces, resulting in poor adhesion [16]. To overcome this weak adhesion, reactive metals such as Cr or Ti are often employed as adhesion layers that enhance the interface characteristics between the electrode metal and the 2D material [17]. Additionally, the work function of Cr (≈4.5 eV) is lower than that of Au (≈5.1 eV), leading to a lower Schottky barrier height (SBH) when Cr is in contact with n–type WSe_2_. In contrast, Au with a higher work function induces stronger band bending and a higher SBH, making electron injection more difficult and increasing contact resistance [18].

While 5 nm and 10 nm adhesion layer thicknesses are commonly employed for the electrodes of WSe_2_ FETs, more comprehensive studies are required to justify their selection and assess their impact on device performance. Given that even nanometer–scale changes at the metal–semiconductor interface can affect charge transport, optimizing the thickness of the Cr adhesion layer is crucial for improving the device characteristics. Furthermore, although adhesion layers have been relatively well studied in devices based on graphene and MoS_2_, their impact on WSe_2_–based devices also warrants investigation due to different material properties, including the work function.

In this study, we investigate the role of Cr adhesion layer thickness in determining contact resistance and SBH in WSe_2_–based FETs. Using the transfer length method (TLM), we first extract contact resistance in devices with Cr thicknesses of 1 nm, 4 nm, and 7 nm. While the 4 nm and 7 nm Cr devices exhibit low contact resistance below 200 kΩ·μm, the 1 nm Cr device shows a significantly higher resistance of 6.6 MΩ·μm. We then conduct temperature–dependent transport measurements and Arrhenius analysis, with the results demonstrating that the SBH decreases as the Cr layer thickness increases. This trend suggests that thinner Cr layers generate higher Schottky barriers, leading to reduced carrier injection efficiency.

## 2. Experimental Methods

**Figure 1:**
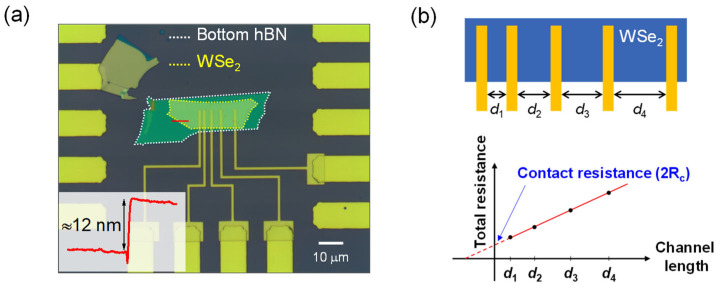
Optical microscopic image and schematic of the transfer length method (TLM). (**a**) Optical microscopic image of a WSe_2_ field–effect transistor (FET). The white and yellow dashed lines mark the bottom hBN and WSe_2_ flake, respectively. Cr/Au electrodes define the channel lengths of 1, 2, 3, and 5 μm, with a fixed channel width of 8 μm. The red line indicates the atomic force microscopy (AFM) scan measuring WSe_2_ thickness. Inset: AFM height profile showing a 12 nm WSe_2_ flake. (**b**) Scheme of the device structure and TLM plot as an example. The x–axis represents the channel length (d_1_–d_4_), and the y–axis shows total resistance. The y–intercept indicates contact resistance.

Figure 1a shows an optical microscopic image of a fabricated WSe_2_ FET device. Multilayer WSe_2_ and hexagonal boron nitride (hBN) were stacked on 300 nm SiO_2_ on a highly p–doped Si substrate using a dry pick–up method with a polypropylene carbonate stamp [19]. Placing hBN under WSe_2_ flakes maintains the intrinsic properties of WSe_2_ and reduces gate–induced hysteresis, which helps systematic electrical measurement [20,21,22]. As shown in the inset of Figure 1a, the thickness of the WSe_2_ was measured to be approximately 12 nm using atomic force microscopy (AFM) (Park systems, Suwon, Republic of Korea). While this image represents a single device, multiple WSe_2_ FETs were fabricated and investigated in this study, using WSe_2_ flakes with thicknesses ranging from 10 to 20 nm. The Au electrodes with a Cr adhesion layer were fabricated using e–beam lithography and e–beam evaporation. To obtain the contact resistance of the fabricated devices using the TLM, the channel length was varied between 1, 2, 3, and 5 μm, while the channel width was fixed at 8 μm. Figure 1b illustrates a schematic of the device structure and a representative plot showing the concept of the TLM to calculate the contact resistance [23]. In this method, we determine the contact resistance from the y–intercept, corresponding to zero channel length, by measuring the total resistance across multiple channels with varying lengths (d_1_ to d_4_). To investigate the effect of Cr thickness on contact resistance, Cr layers of 1, 4, or 7 nm were deposited as adhesion layers before depositing the Au layers of identical 50 nm thickness. Because the WSe_2_–metal interface critically influences the contact resistance, the Cr adhesion metal layer in contact with the WSe_2_ flake was deposited at a slower deposition rate, with the aim to reduce damage to the 2D material surface during the deposition process (0.1 Å/s for Cr and 0.5 Å/s for Au). The electrical characteristics of all devices were measured using a Keithley 2502 picoammeter (Keithley Instrument, Cleveland, OH, USA), with the gate voltage (V_G_) applied via a back–gate configuration. All measurements were conducted under vacuum conditions to reduce the effects of surface contaminations due to ambient molecules [22,24].

## 3. Results and Discussion

### 3.1. Calculation of Contact Resistance Using TLM

**Figure 2:**
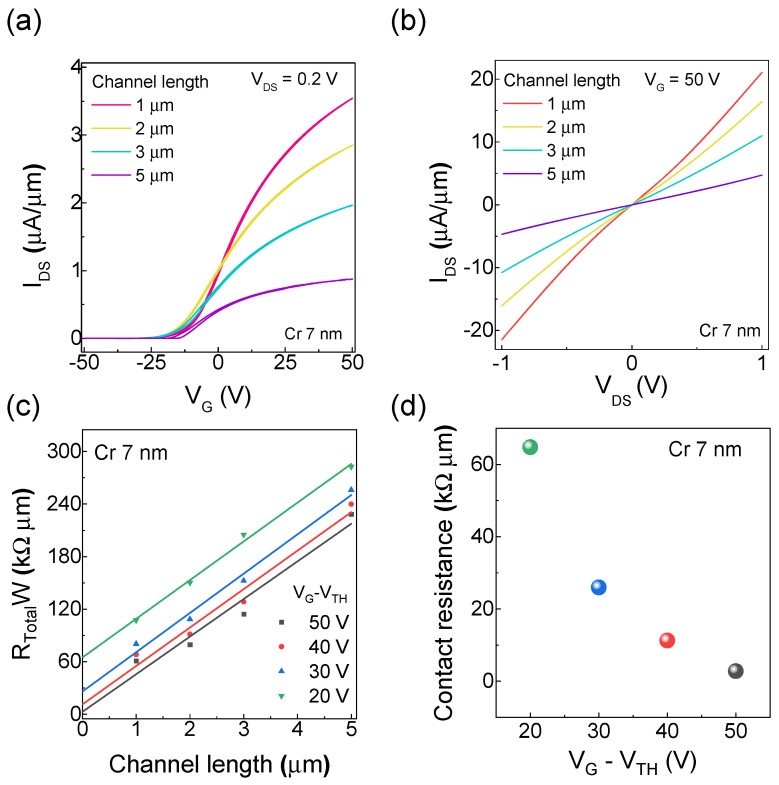
Channel length–dependent electrical characteristics of a WSe_2_ FET with 7 nm Cr thickness. (**a**) Transfer curves at a drain–source voltage (*V_DS_*) of 0.2 V. (**b**) Output curves at a gate voltage (V_G_) of 50 V. The drain–source current (*I_DS_*) is normalized by the channel width. (**c**) TLM plots showing contact resistance at various doping concentrations, calculated as V_G_ − V_TH_, where V_TH_ is the threshold voltage. (**d**) Contact resistance as a function of doping concentration.

Figure 2a,b show the transfer and output curves of a WSe_2_ FET with varying channel lengths with a fixed Cr thickness of 7 nm. Here, V_G_ was swept from −50 V to +50 V with the drain–source voltage (*V_DS_*) fixed at 0.2 V. The observed n–type behavior is consistent with a previous report using Cr/Au electrodes [25]. As shown in Figure 2c,d, the drain–source current (*I_DS_*) decreases with increasing channel length due to general resistive properties, which allows for the calculation of the contact resistance using the TLM [23]. Notably, a substantial reduction in contact resistance was observed at higher doping concentrations. This reduction is attributed to a decrease in the width of the depletion layer near the metal–semiconductor interface, which reduces the width of the barrier for tunneling [26,27]. As a thinner tunneling barrier allows a higher current to flow, the contact resistance decreases.

**Figure 3:**
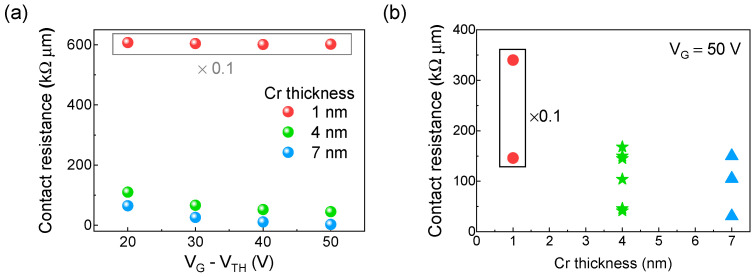
Effect of Cr adhesion layer thickness on contact resistance. (**a**) Contact resistance as a function of doping concentration for devices with different Cr thicknesses. (**b**) Summary of contact resistance across multiple devices with varying Cr thickness, at V_G_ = 50 V. In both panels, the drain–source voltage is fixed at 0.2 V for 4 nm and 7 nm Cr, and at 1 V for 1 nm Cr due to low current levels. The values for 1 nm Cr are scaled by 0.1 for comparison.

To investigate the role of the adhesion metal layer thickness in the contact resistance, we obtained the transfer curves of WSe_2_ FETs with Cr thicknesses of 1, 4, and 7 nm and extracted the corresponding contact resistance, as shown in Figure 3a. We observe significantly higher contact resistance in the device with a Cr thickness of 1 nm, reaching ≈6.6 MΩ∙μm at V_G_ − V_TH_ = 50 V. In comparison, the device with 4 nm Cr shows a markedly lower contact resistance of ≈45 kΩ∙μm under the same doping concentration, representing a reduction by a factor of ≈150. The 7 nm Cr device also shows a resistance much lower than that of the 1 nm device, indicating that dramatic improvement occurs once the Cr thickness reaches 4 nm. Analyzing the dependence of contact resistance on doping concentration for different Cr thicknesses, we consistently observe that higher doping levels result in lower contact resistance independently of Cr thickness. Figure 3b plots the contact resistances at V_G_ = 50 V for several devices fabricated under the same conditions, where the resistance variation by thickness originates from unintended fabrication variation. The contact resistance for a Cr thickness of 1 nm remains in the megaohm range, while in contrast, the contact resistances for Cr thicknesses of 4 nm and 7 nm are below 200 kΩ∙μm. These results indicate that a Cr thickness of at least 4 nm is required to achieve efficient n–type carrier injection in WSe_2_ FETs.

### 3.2. Schottky Barrier Height by Cr Thickness

The contact resistance in these FETs is strongly influenced by the interface between WSe_2_ and Cr. When WSe_2_ and Cr come into contact, Fermi level alignment according to the difference in their Fermi levels leads to band bending, which in turn forms a Schottky barrier [18]. To contribute to the drain–source current, charge carriers must overcome the Schottky barrier via either thermionic emission or tunneling [28]. In order to find the relation between the contact resistance and the Schottky barrier as a function of Cr thickness, we extracted the SBH from temperature–dependent resistance measurements of WSe_2_ FETs with varying Cr adhesion layer thickness.

According to the Arrhenius equation, the current density changes with temperature under the following equation,

(1) $$J=A^{**}T^{2}\exp\left(-\frac{q\phi_{B}}{k_{B}T}\right)\left[\exp\left(\frac{qV_{DS}}{k_{B}T}\right)-1\right]$$

Here, J is the drain–source current density, $A^{**}$ is the Richardson constant, $k_{B}$ is the Boltzmann constant, and $\phi_{B}$ is the Schottky barrier height. Taking the logarithm of both sides of the equation, we get:

(2) $$\ln\left(\frac{I_{DS}}{T^{2}}\right)=\ln\left({AA}^{**}\right)-\frac{q(\phi_{B}-V_{DS})}{k_{B}T}$$

**Figure 4:**
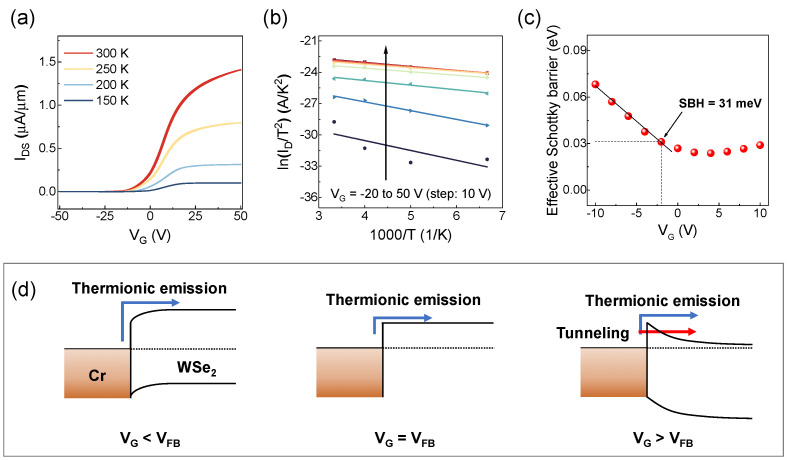
Extraction of Schottky barrier height (SBH) from temperature–dependent transfer curves of a device with a 7 nm Cr adhesion layer and 5 μm channel length. (**a**) Transfer curves measured at various temperatures with *V_DS_* = 0.2 V. (**b**) Arrhenius plots for gate voltages ranging from −20 V to +50 V. (**c**) Effective Schottky barrier as a function of gate voltage. The SBH is determined as the point where linearity ends (black arrow). (**d**) Schematic energy band diagrams of the Cr–WSe_2_ interface at different gate voltages in relation to the flat–band voltage (V_FB_).

Figure 4a presents the temperature–dependent transfer curves of WSe_2_ FETs with a 7 nm Cr layer. We observe an increase in the on–current with temperature, implying enhanced thermionic emission over the Schottky barrier at elevated temperatures, thereby improving carrier injection at the metal-semiconductor interface. These transfer curves were then used to generate Arrhenius plots of $\ln\left(\frac{I_{DS}}{T^{2}}\right)$ versus 1000/T for the WSe_2_ FETs, as shown in Figure 4b, with gate voltages ranging from −20 V to +50 V in 10 V steps. Steeper slopes at negative gate voltages indicate stronger temperature dependence, while flatter slopes at higher gate voltages suggest less dominant thermal dependence and possible contribution by tunneling. By extracting the slope at each gate voltage in the Arrhenius plots, the effective SBH can be obtained as a function of V_G_, which is plotted in Figure 4c. In this plot, the effective SBH initially varies linearly at negative gate voltage, but deviates from linearity beyond a certain point, as marked with the black arrow. This transition point is used to determine the SBH [28]. For the device with 7 nm Cr, the extracted SBH is ≈31 meV. In order to explain the transition point in more detail, in Figure 4d we present simplified band diagrams for gate voltages below, at, and above the flat–band voltage (V_FB_), referring to the gate voltage at which the band flattens in the semiconductor region. When V_G_ is less than V_FB_, thermionic emission dominates the electron transport owing to the large Schottky barrier width, resulting in the effective barrier height scaling linearly with V_G_. However, when V_G_ is higher than V_FB_, tunneling begins to contribute via the narrower Schottky barrier width from increased doping concentration, breaking the linear relationship between the effective barrier height and the gate voltage [29,30]. Therefore, the point at which linearity ends in the graph of effective Schottky barrier versus gate voltage can be considered as the SBH.

**Figure 5:**
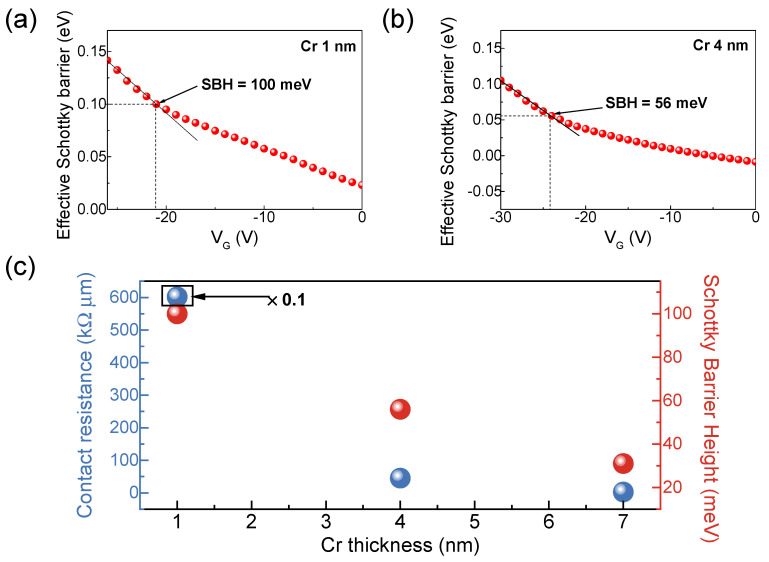
Extraction of SBH from temperature–dependent transfer curves of a device with 1 nm and 4 nm Cr adhesion layers. (**a**,**b**) Effective Schottky barrier versus gate voltage for 1 nm and 4 nm Cr, respectively. Transition points are marked with the black arrows. Measurements were taken at *V_DS_* = 1 V. (**c**) Contact resistance and SBH as a function of Cr thickness. The contact resistance for 1 nm Cr is scaled by 0.1 for comparison.

Additionally, we extracted the SBH for Cr thicknesses of 1 nm and 4 nm and present the results in Figure 5a and Figure 5b, respectively. Figure 5c summarizes the contact resistance and SBH as a function of Cr thickness in fabricated WSe_2_ FETs. As the Cr thickness increases, the SBH decreases from 100 meV at 1 nm to 56 meV at 4 nm. Both contact resistance and barrier height decrease with increasing Cr thickness. These experimental results confirm that the higher contact resistance observed with the thin Cr adhesion layer (1 nm) can be attributed to the increased SBH.

To validate the SBH extraction methodology used in this study, next we investigated the variation in SBH induced by image force lowering. Near a metal-semiconductor interface, electrons or holes experience additional attractive forces from their induced image charges, which reduces the potential energy barrier and facilitates transport. This phenomenon, known as image force lowering, decreases the effective SBH [31,32,33,34]. The barrier height decreased by this effect is determined by the following equation:

(3) $$∆\phi_{B}=\sqrt{\frac{qE}{4\pi \varepsilon_{s}}}$$

where *q* is the charge, *ε_s_* is the dielectric constant of the semiconductor, and *E* is the electric field inside the semiconductor. As *V_DS_* increases, the electric field *E* increases, and accordingly, Δ*ϕ_B_* increases, and the effective barrier height Δ*ϕ_B_^eff^* that the electrons must overcome is lowered according to

(4) $$\phi_{B}^{eff}=\phi_{B}-{∆\phi }_{B}$$

**Figure 6:**
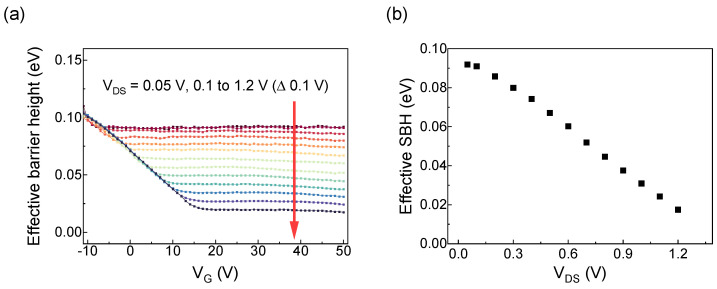
(**a**) Effective SBH of a 1 nm Cr device measured at various drain–source voltages: 0.05 V, and from 0.1 V to 1.2 V in 0.1 V steps. The channel length is 3 μm. (**b**) Effective SBH versus drain–source voltage to verify image force lowering.

Figure 6a shows the change in the effective SBH for the 1 nm Cr device at a drain–source voltage of 0.05 V and as it increases from 0.1 V to 1.2 V in 0.1 V steps. The effective barrier height decreases nearly linearly with increasing *V_DS_*, consistent with the image force lowering effect. In Figure 6b, we plot the effective SBH versus *V_DS_*. The results show that the effective SBH decreases as *V_DS_* increases, providing evidence that the reduction in SBH is due to the image force lowering effect caused by the increased electric field inside the semiconductor.

## 4. Conclusions

In summary, we showed the effect of Cr adhesion layer thickness on the contact resistance and Schottky barrier characteristics of WSe_2_–based FETs. Using a device structure compatible with the TLM, we first extracted the contact resistance as a function of Cr layer thickness. While devices with 4 nm and 7 nm Cr layers exhibited low contact resistance below 200 kΩ·μm, the device with a 1 nm Cr layer showed a significantly higher resistance of 6.6 MΩ·μm, indicating that Cr thickness critically affects the quality of the metal–semiconductor interface. Therefore, we conclude that Cr thicknesses of 4 nm and above represent the optimum thickness for minimizing contact resistance in FET devices. In addition, we extracted the SBH from temperature–dependent measurements using Arrhenius plots. The extracted SBHs were 100 meV for 1 nm Cr, 56 meV for 4 nm, and 31 meV for 7 nm. This trend supports that thinner Cr layers result in higher Schottky barriers, leading to poor carrier injection and increased contact resistance. Furthermore, we examined the image force lowering effect and found that the SBH decreased linearly with increasing drain–source voltage, confirming the theoretical predictions and the reliability of our SBH results. Our findings demonstrate that optimizing the Cr adhesion layer thickness is essential for minimizing contact resistance and improving device performance in WSe_2_–based FETs. These insights offer valuable guidelines for the design of low-resistance contacts in 2D material–based electronic devices.

## Data Availability

Data is contained within the article.
